# Evaluating Rare Amino Acid Substitutions (RGC_CAMs) in a Yeast Model Clade

**DOI:** 10.1371/journal.pone.0092213

**Published:** 2014-03-17

**Authors:** Kenneth Polzin, Antonis Rokas

**Affiliations:** Department of Biological Sciences, Vanderbilt University, Nashville, Tennessee, United States of America; National Center for Biotechnology Information, United States of America

## Abstract

When inferring phylogenetic relationships, not all sites in a sequence alignment are equally informative. One recently proposed approach that takes advantage of this inequality relies on sites that contain amino acids whose replacement requires multiple substitutions. Identifying these so-called RGC_CAM substitutions (after Rare Genomic Changes as Conserved Amino acids-Multiple substitutions) requires that, first, at any given site in the amino acid sequence alignment, there must be a minimum of two different amino acids; second, each amino acid must be present in at least two taxa; and third, the amino acids must require a minimum of two nucleotide substitutions to replace each other. Although theory suggests that RGC_CAM substitutions are expected to be rare and less likely to be homoplastic, the informativeness of RGC_CAM substitutions has not been extensively evaluated in biological data sets. We investigated the quality of RGC_CAM substitutions by examining their degree of homoplasy and internode certainty in nearly 2.7 million aligned amino acid sites from 5,261 proteins from five species belonging to the yeast *Saccharomyces* sensu stricto clade whose phylogeny is well-established. We identified 2,647 sites containing RGC_CAM substitutions, a number that contrasts sharply with the 100,887 sites containing RGC_non-CAM substitutions (i.e., changes between amino acids that require only a single nucleotide substitution). We found that RGC_CAM substitutions had significantly lower homoplasy than RGC_non-CAM ones; specifically RGC_CAM substitutions showed a per-site average homoplasy index of 0.100, whereas RGC_non-CAM substitutions had a homoplasy index of 0.215. Internode certainty values were also higher for sites containing RGC_CAM substitutions than for RGC_non-CAM ones. These results suggest that RGC_CAM substitutions possess a strong phylogenetic signal and are useful markers for phylogenetic inference despite their rarity.

## Introduction

Recent advances in genomics, computer science and systematics theory have invigorated the pursuit of assembling the tree of life [1]. Understanding what information can be gleaned from the available molecular sequences is vital for constructing as well as for ensuring the accuracy of the tree of life. A major hindrance to the reconstruction of evolutionary relationships is homoplasy, which is in the context of nucleotide or amino acid sequence alignment data can be defined as the independent evolution of the same character state in two or more taxa [2], [3]. Two parallel and complementary approaches for tackling homoplasy have been developed. On the one hand, several different phylogenetic algorithms and models of sequence evolution have been developed to reduce the impact of homoplasy on phylogenetic reconstruction [4]. For example, every model of amino acid sequence evolution is a weighted substitution matrix of the 190 possible substitutions possible between the 20 amino acids [5], which means that certain amino acid substitutions carry more weight in phylogenetic inference than others. On the other hand, there has been a renewed focus on identifying very rare molecular changes, so-called rare genomic changes (RGCs), that have low homoplasy and a clear phylogenetic signal [6]. RGC examples include insertions or deletions [7], [8], retroposon integrations [9], changes in gene order [10], and gene duplications and losses [11].

It was recently proposed that conserved amino acids whose replacement requires multiple substitutions, known as RGC_CAM substitutions, represent a novel type of rare genomic change [12]. Aimed at reducing the impact of homoplasy on phylogenetic inference, RGC_CAM substitutions are typically identified only by considering sites in an amino acid sequence alignment that contain amino acid residues that require 2 or 3 nucleotide substitutions to replace one another. Because replacements between amino acids that require two or more nucleotide substitutions are less likely than replacements that require a single nucleotide substitution, which we term RGC_non-CAM substitutions, RGC_CAM substitutions are expected to exhibit lower homoplasy [13]. RGC_CAM substitutions were first used to arbitrate on a vexing controversy of the phylogeny of animal phyla [12]–[15]; specifically, they were used in deciding whether phylogenetic evidence supported the existence of a clade comprised of phyla that undergo molting (this is known as the Ecdysozoa clade and is thought to include Arthropods, Nematodes and other lineages thought to undergo molting or ecdysis) [16] or, alternatively, the existence of a clade comprised of phyla that share a true body cavity (this is known as the Coelomata clade and is thought to include Arthropods and Chordates, but not Nematodes). The use of RGC_CAM substitutions did not settle the controversy, as support for the two alternative hypotheses appears to be sensitive to choice and number of taxa used in the analyses [12]–[15].

While RGC_CAM substitutions have been unable to resolve these controversial and much debated relationships between metazoan phyla, this does not necessarily mean that their quality is not as good as, or better than standard amino acid substitutions [see also Ref. 13]. To further evaluate the informativeness and utility of RGC_CAM substitutions, we identified and examined all 2,647 RGC_CAM substitutions present in amino acid sequence alignments from 5,261 orthologous groups of proteins obtained from five *Saccharomyces* sensu stricto species that possess a well-resolved phylogeny: *Saccharomyces cerevisiae*, *Saccharomyces paradoxus*, *Saccharomyces mikatae*, *Saccharomyces kudriavzevii*, and *Saccharomyces bayanus* [17]. Our analyses of the yeast RGC_CAM substitutions suggest that they exhibit significantly lower homoplasy and higher internode certainty that the 100,887 RGC_non-CAM substitutions observed in the same dataset. Thus, even though RGC_CAM substitutions appear to be extremely rare, our analyses suggest that they are highly informative markers of phylogeny.

## Methods

### Definition of RGC_CAM Substitutions and Search Implementation

**Figure 1:**
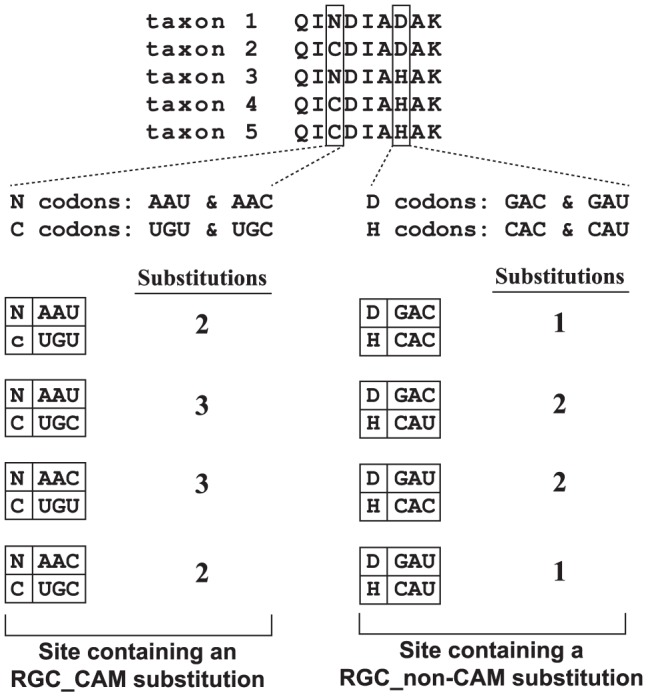
Identification of sites containing RGC_CAM and RGC_non-CAM substitutions. Rare genomic changes in sites containing amino acids with multiple substitutions (RGC_CAM substitutions) occur at sites in aligned amino acid sequences where: 1) a minimum of two different amino acids are present, 2) each amino acid is present in at least two species at that site, and 3) the amino acids present require two or more nucleotide substitutions to change from one into another. For example, the third site in this sequence alignment meets these three criteria to be considered as containing a RGC_CAM substitution; it contains two amino acids, arginine (N) and cysteine (C), each of which are present in at least two species, whose replacement with one another requires at least two nucleotide substitutions for any combination of codons encoding for the two amino acids. In contrast, the seventh site in this alignment is a site that contains a RGC_non-CAM substitution; this site meets the first two criteria–it contains two amino acids, aspartic acid (D) and histidine (H), each of which are present in at least two species–but does not meet the third since a minimum of one nucleotide substitution is required to replace aspartic acid (D) with histidine (H) and vice versa.

We define RGC_CAM substitutions as substitutions that occur at a site in an amino acid sequence alignment that fulfill three criteria: a) a minimum of two different amino acids must be present at the site, b) each amino acid must be present in at least two taxa, and c) a replacement of one amino acid for the other must take a minimum of two nucleotide substitutions (Fig. 1). Determination of the minimum number of nucleotide changes is done by considering all possible codons for the amino acids present at a site and not by directly examining the nucleotide sequences of the species studied. Note that this definition of RGC_CAMs relaxes the original definition of Rogozin and co-workers [12] by replacing their requirement that one of the amino acid residues is assumed to represent the ancestral character state with the requirement that each amino acid present at a site must be present in at least two species.

A Perl script (list_rgc_cams.pl) was written to use the standard genetic code table and systematically compare the codons of each amino acid to the codons of every other amino acid. Doing so created a matrix of all the amino acid pairs that require a minimum of two nucleotides to change from one amino acid to the other, which we name RGC_CAM substitution matrix (Table S1). A second Perl script (rgc_cams.pl) was written that takes amino acid FASTA files as input and uses the RGC_CAM substitution matrix to find and output all sites that contain RGC_CAM substitutions in a new FASTA file. For comparison purposes, a complementary Perl script (nonrgc_cams.pl) was written to identify all sites in an amino acid sequence alignment that meet the first two RGC_CAM substitution criteria (i.e., a minimum of two different amino acids must be present at the site, and each amino acid must be present in at least two taxa) but in which the substitution of one amino acid for the other occurs via a single nucleotide substitution. The three scripts, alongside with example FASTA files and documentation are available through the Rokas lab website (http://as.vanderbilt.edu/rokaslab/data/Polzin_Rokas_PLoSONE_2014.zip).

### The *Saccharomyces* sensu stricto Dataset

The amino acid alignments from the 5,261 high-quality groups of orthologous proteins across five species in the genus *Saccharomyces* sensu stricto genus were obtained from www.SaccharomycesSensuStricto.org [17]. Sites containing RGC_CAM substitutions and RGC_non-CAM substitutions were identified by examining all 2,668,077 sites of the 5,261 protein sequence alignments using the three Perl scripts described in the previous section. The resulting FASTA files containing sites that exhibit RGC_CAM substitutions and, separately, RGC_non-CAM substitutions were analyzed.

### Phylogenetic Analysis

To measure the prevalence of sites containing RGC_CAM substitutions and of specific amino acids, we calculated the ratio of RGC_CAM sites to the full *Saccharomyces* sensu stricto sequence length, the number of proteins containing RGC_CAM sites, and the number of RGC_CAM sites per protein. We also determined the frequency with which amino acids were present with specific other amino acids at sites along the sequences.

Phylogenetic analyses were performed using parsimony analysis on the RGC_CAM and RGC_non-CAM datasets using PAUP* 4.10b [18]. Robustness in inference was assessed using bootstrap resampling [19]. Internode Certainty (IC), Internode Certainty All (ICA), Tree Certainty (TC), and Tree Certainty All (TCA) were calculated in RAxML 7.9.1 for the RGC_CAM and RGC_non-CAM datasets [20], [21]. Each site that contains a RGC_CAM or a RGC_non-CAM substitution defines a bipartition that splits the 5 yeast species into two groups. The bipartitions defined from all RGC_CAM and RGC_non-CAM sites were merged into single files and RAxML was used to calculate IC, ICA, TC and TCA values on the yeast phylogeny [20], [22]. Briefly, IC quantifies the degree of incongruence for a given bipartition defined by a site across a set of sites by considering the number of sites supporting that bipartition jointly with the number of sites supporting the most prevalent bipartition that conflicts with it (IC) or jointly with the numbers of sites supporting all the most prevalent bipartitions that conflict with it (ICA). TC and TCA represent the sums of the IC and ICA values, respectively from all internodes in the tree set [20], [22].

The degree of homoplasy in RGC_CAM and RGC_non-CAM sites was measured using the homoplasy index (HI) [23]. Briefly, the HI is 1 minus the consistency index, which in the context of this study is the ratio of the minimum number of changes a site exhibits on the yeast phylogeny divided by the minimum number of changes a site exhibits on any phylogenetic tree. HI values range from 0 to 1, with values closer to 0 indicating a very low degree of homoplasy and with values closer to 1 indicating a very high degree of homoplasy. HI calculation on any site with an RGC_CAM or RGC_non-CAM substitution on a 5-taxon fully resolved unrooted tree can take only one of two values (0 or 0.5). Thus, the resulting data can form a 2 x 2 contingency table containing RGC_CAM sites with HI  =  0 (in support of the yeast phylogeny), RGC_CAM sites with HI  = 0.5 (in contradiction to the yeast phylogeny), RGC_non-CAM sites with HI  =  0, and RGC_non-CAM sites with HI  =  0.5. We tested whether the two types of sites differed in their degree of homoplasy using Fisher’s exact test.

To visualize the comparison of the distribution of HI values between RGC_CAM sites and RGC_non-CAM sites, we also calculated HI on 52 bins of 50 RGC_CAM sites, which were generated by randomly distributing the 2,647 RGC_CAM sites (52 × 50 = 2,600, which means that all but 47 RGC_CAM sites were used in these calculations). To compare these 52 bins of RGC_CAM sites, we generated 52 equally sized bins of randomly picked RGC_non-CAM sites. For each bin, the mean HI was calculated and used in a Wilcoxon Rank-Sum test to determine if the HI values of RGC_CAM site bins and RGC_non-CAM site bins were significantly different.

## Results and Discussion

Examination of 2,668,077 columns of amino acid sequence alignment from five *Saccharomyces* species resulted in the identification of 2,647 sites containing RGC_CAM substitutions and 100,887 sites containing RGC_non-CAM substitutions; thus, approximately 0.1% and 3.8% of sites in the alignment contain RGC_CAM and RGC_non-CAM substitutions, respectively. Although sites containing RGC_CAM substitutions are found in 1,723 protein alignments, 1,142 of them contain only one RGC_CAM site, with the remaining 581 protein alignments containing two or more. In contrast, 5,079 of the 5,261 protein alignments (∼96.5%) contain one or more RGC_non-CAM sites. Interestingly, the protein alignments for the genes YDL081C, YGL123W, YLR044C, YMR260C, YMR290C, YNL190W, YNL090W, and YPL025C contained one or two RGC_CAM sites but no RGC_non-CAM ones. These results show that RGC_CAM substitutions comprise a very small fraction of the total number of informative sites in protein sequence alignment data, suggesting that they may be of practical use only when large amounts of sequence data are available.

**Figure 2:**
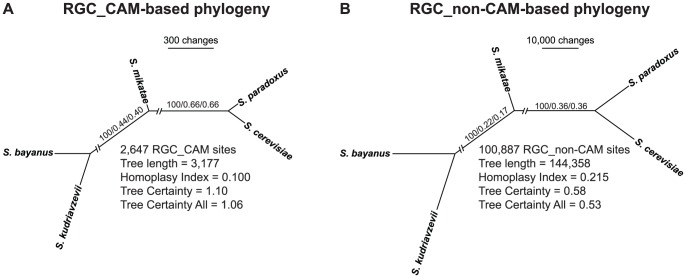
Phylogenies of *Saccharomyces* sensu stricto species reconstructed using only sites that contain RGC_CAM and RGC_non-CAM substitutions. Unrooted phylogenies of *Saccharomyces* sensu stricto species constructed using the 2,647 RGC_CAM sites (panel A) and the 100,887 RGC_non-CAM sites (B) using the parsimony optimality criterion. Values above internodes represent percentage bootstrap support, internode certainty (IC), and internode certainty all (ICA). The average Homoplasy Index per site, tree certainty (TC) and tree certainty all (TCA) are also shown for the two data matrices. Note that sites containing RGC_CAM substitutions have substantially higher incongruence and substantially lower homoplasy than RGC_non-CAM sites. Finally, note that tree lengths can also calculated from the data shown in Table 1. Because tree length  =  (n–m) + 2*m, where m  =  homoplastic sites and n  =  total sites, for RGC_CAM sites we have m = 530 sites and n = 2,647 sites. Thus, the tree length for the RGC_CAM data matrix equals (2,647–530) + 2*530 = 3,177.

Phylogenetic analysis of both RGC_CAM and RGC_non-CAM sites recovered the established species phylogeny (Fig. 2) [20], [24], [25] with absolute support, as the two internodes received 100% bootstrap values in both analyses. Evaluation of RGC_CAM and RGC_non-CAM sites with the recently developed Internode Certainty and Tree Certainty metrics [20] showed that their values were substantially higher for RGC_CAM substitutions than for RGC_non-CAM ones, indicating that RGC_CAM sites are substantially more congruent in their phylogenetic signal than RGC_non-CAM sites. Specifically, IC/ICA values were 0.66/0.66 for RGC_CAM sites but only 0.36/0.36 for RGC_non-CAM sites for the clade that groups *S. cerevisiae* and *S. paradoxus*. Focusing on the IC metric, a value of 0.66 means that the most prevalent grouping of *S. cerevisiae* and *S. paradoxus* received much more support than the next most well-supported, but conflicting, grouping of *S. cerevisiae* and *S. bayanus*; specifically, the *S. cerevisiae – S. paradoxus* grouping was supported by 937 RGC_CAM sites whereas the *S. cerevisiae – S. bayanus* grouping was supported by only 62 RGC_CAM sites (a greater than 9:1 ratio). For RGC_non-CAM sites, the IC value of 0.36 for the same *S. cerevisiae – S. paradoxus* grouping stems from the fact that there was much larger support for a conflicting grouping; specifically, whereas the *S. cerevisiae – S. paradoxus* grouping was supported by 25,625 RGC_non-CAM sites, the *S. cerevisiae – S. bayanus* grouping was supported by 5,008 RGC_non-CAM sites (approximately a 5:1 ratio). Similarly, IC/ICA values were 0.44/0.40 for RGC_CAM sites but only 0.22/0.17 for RGC_non-CAM sites for the clade that groups *S. cerevisiae*, *S. paradoxus* and *S. mikatae*. Thus, the TC/TCA values for the phylogeny constructed based on RGC_CAM substitutions were 1.10/1.06 whereas those for the phylogeny constructed based on RGC_non-CAM substitutions were 0.58/0.53.

To further investigate the degree of homoplasy in sites containing RGC_CAM and RGC_non-CAM substitutions, we calculated the average HI per site across the two data sets. The mean HI per site was 0.100 for RGC_CAM sites and 0.215 for RGC_non-CAM sites. We also measured the numbers of RGC_CAM sites that support the accepted yeast phylogeny (2,117 sites), of RGC_CAM sites that do not support the yeast phylogeny (530), as well as the numbers of RGC_non-CAM sites that support (57,416), or do not support (43,471) the yeast phylogeny (Table 1). A Fisher’s exact test on the resulting 2 x 2 table indicates that the difference in the degree of support for the yeast phylogeny between RGC_CAM and RGC_non-CAM sites is significant (*p*-value < 2.2×10^−16^).

**Table 1:** Support of various bipartitions from RGC_CAM and RGC_non-CAM sites.

Bipartition∧	Bipartition name	Number of RGC_CAM sites that support bipartition	% of RGC_CAM sites that support bipartition	Number of RGC_non-CAM sites that support bipartition	% of RGC_non-CAM sites that support bipartition
*Bipartitions present in the yeast phylogeny*					
((c,p,m),(k,b))	kb	1,180	44.6%	31,791	31.5%
((k,b,m),(c,p))	cp	937	35.4%	25,625	25.4%
	Total:	2,117	80.0%	57,416	56.9%
*Bipartitions that conflict with the yeast phylogeny*					
((c,k,p),(b,m))	bm	177	6.7%	9,520	9.4%
((c,b,p),(k,m))	km	141	5.3%	8,756	8.7%
((k,p,m),(c,b))	cb	62	2.3%	5,008	5.0%
((k,b,p),(c,m))	cm	42	1.6%	4,934	4.9%
((c,k,b),(p,m))	pm	42	1.6%	4,817	4.8%
((c,k,m),(b,p))	bp	29	1.1%	3,623	3.6%
((b,p,m),(c,k))	ck	26	1.0%	3,736	3.7%
((c,b,m),(k,p))	kp	11	0.4%	3,077	3.0%
	Total:	530	20.0%	43,471	43.1%

**∧**Key: c  =  *S. cerevisiae*, p  =  *S. paradoxus*, m  =  *S. mikatae*, k  =  *S. kudriavzevii*, and b  =  *S. bayanus*; each bipartition is named after the group formed by two of the five species. Analysis of RGC_CAMs and RGC_non-CAMs in five species means that each informative site with contain an amino acid character shared by three species and a different amino acid character shared by the other two species. Because there are 10 possible bipartitions for an unrooted tree from 5 species, this means that each informative site will support one of ten bipartitions. Given that the accepted unrooted phylogeny is ((c,p),m,(k,b)), each of the ten bipartitions either support it by grouping (c,p) and (k,b) (as the “kb” and “cp” bipartitions do) or contradict it by separating those species (as the other bipartitions do). Thus the percentage of RGC_CAM sites that support the accepted yeast phylogeny is 80.0% (kb + cp) while the RGC_non-CAM support is only 56.9%.

**Figure 3:**
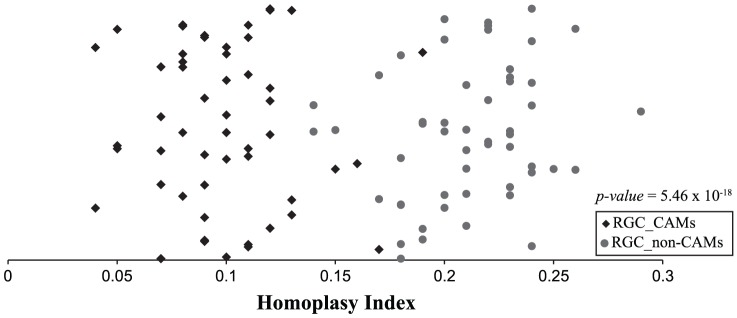
Distribution of homoplasy index values in 52 bins containing 50 RGC_CAM substitutions and in 52 bins containing 50 RGC_non-CAM substitutions. Homoplasy index (HI) values were calculated on 52 bins of 50 RGC_CAM sites, which were generated by randomly distributing the 2,647 RGC_CAM sites (52×50 = 2,600, which means that all but 47 RGC_CAM sites were used in these calculations). To compare these 52 bins of RGC_CAM sites, 52 equally sized bins of randomly picked RGC_non-CAM sites were also generated. The average HI of each bin is plotted on the X axis; for the Y axis, a random number between 0 and 1 was assigned to each bin to aid visualization. Average HI values of bins containing RGC_CAM substitutions are shown as diamonds and average HI values of bins containing RGC_non-CAM substitutions are shown as circles. Note that multiplying the HI value by 100 for each bin shown on the figure provides the number of sites that do not support the tree. For example, for a diamond with HI  =  0.05, this means that 5 RGC_CAM sites do not support the species phylogeny, whereas the other 45 support it. A Wilcoxon Rank-Sum test showed significant difference in the average HI between the RGC_CAM-containing and RGC_non-CAM-containing bins (*p-value*  = 5.46×10^−18^).

To visualize the difference in the degree of homoplasy between the two types of sites, we compared the average HI values between the 52 bins of 50 RGC_CAM sites against all 2,017 equally sized RGC_non-CAM bins as well as against 52 RGC_CAM HI bins (Fig. 3). This comparison showed highly significant differences in the degree of homoplasy present in RGC_CAM versus RGC_non-CAM sites (Two-tailed Wilcoxon Rank-Sum tests: 52 RGC_CAM bins versus 2,017 RGC_non-CAM bins: *p-value*  =  1.55×10^−33^; 52 bins versus 52 RGC_non-CAM bins: *p-value*  =  5.46×10^−18^) (Fig. 3). The lower degree of homoplasy present in RGC_CAM sites relative to RGC_non-CAM ones is consistent with the higher degree of congruence identified by the Internode Certainty-based analyses (Fig. 2), suggesting that in so far as congruence and low homoplasy are concerned, RGC_CAM sites are likely better markers for phylogenetic inference.

In conclusion, we have developed computational scripts that allowed us to examine nearly 2.7 million sites of amino acid alignment from 5 closely related *Saccharomyces* yeast species and identify 2,647 sites that contain RGC_CAM substitutions and 100,887 sites that contain RGC_non-CAM substitutions. Comparison of the congruence (by means of examining internode certainty) and homoplasy (by means of examining the homoplasy index) of RGC_CAM and RGC_non-CAM sites indicated that RGC_CAM substitutions show much higher levels of congruence and significantly lower levels of homoplasy relative to RGC_non-CAM substitutions, although it should be noted that both types of markers are able to accurately resolve the phylogeny of these five species. Our results suggest that, although very rare, RGC_CAM substitutions are high quality markers for phylogenetic inference and might be a very attractive source of alternative markers in large datasets showing high levels of homoplasy.

## Supporting Information

Table S1 **RGC_CAM substitution matrix.**
(PDF)
