# Supplementary material for: Evaluating Rare Amino Acid Substitutions (RGC_CAMs) in a Yeast Model Clade
Source: PLoS One. 2014 Mar 17;9(3):e92213. doi: 10.1371/journal.pone.0092213 (PMC3956930; doi:10.1371/journal.pone.0092213)
Supplement: Table S1 — RGC_CAM substitution matrix. (PDF) [file pone.0092213.s001.pdf]

Table S1: RGC_CAM substitution matrix. Positions marked with an X represent RGC_CAM substitutions and differ by at least two nucleotides

	A	R	N	D	C	Q	E	G	H	I	L	K	M	F	P	S	T	W	Y	V	B	Z
**A**		X	X		X	X			X	X	X	X	X	X				X	X			
**R**	X		X	X			X							X					X	X	X	
**N**	X	X			X	X	X	X			X		X	X	X			X		X		X
**D**		X			X	X				X	X	X	X	X	X	X	X	X				
**C**	X		X	X		X	X		X	X	X	X	X		X		X			X	X	X
**Q**	X		X	X	X			X		X			X	X		X	X	X	X	X	X	
**E**		X	X		X				X	X	X		X	X	X	X	X	X	X			
**G**			X			X			X	X	X	X	X	X	X		X		X			
**H**	X				X		X	X		X		X	X	X		X	X	X		X		
**I**	X			X	X	X	X	X	X						X			X	X			X
**L**	X		X	X	X		X	X				X					X		X		X	
**K**	X			X	X			X	X		X			X	X	X		X	X	X		
**M**	X		X	X	X	X	X	X	X					X	X	X		X	X		X	X
**F**	X	X	X	X		X	X	X	X			X	X		X		X	X			X	X
**P**			X	X	X		X	X		X		X	X	X				X	X	X	X	
**S**				X		X	X		X			X	X							X		X
**T**				X	X	X	X	X	X		X			X				X	X	X		X
**W**	X		X	X		X	X		X	X		X	X	X	X		X		X	X	X	X
**Y**	X	X				X	X	X		X	X	X	X		X		X	X		X		X
**V**		X	X		X	X			X			X			X	X	X	X	X			
**B**		X			X	X					X		X	X	X			X				
**Z**			X		X					X			X	X		X	X	X	X			
